# Irrelevant tactile stimulation biases visual exploration in external coordinates

**DOI:** 10.1038/srep10664

**Published:** 2015-05-29

**Authors:** José P. Ossandón, Peter König, Tobias Heed

**Affiliations:** 1 Institute of Cognitive Science, University of Osnabrück, Osnabrück, Germany; 2 Department of Neurophysiology and Pathophysiology, University Medical Center Hamburg-Eppendorf, Martinistr. 52, 20246 Hamburg, Germany; 3 Biological Psychology & Neuropsychology, Faculty of Psychology & Movement Science, University of Hamburg, Hamburg, Germany

## Abstract

We evaluated the effect of irrelevant tactile stimulation on humans’ free-viewing behavior during the exploration of complex static scenes. Specifically, we address the questions of (1) whether task-irrelevant tactile stimulation presented to subjects’ hands can guide visual selection during free viewing; (2) whether tactile stimulation can modulate visual exploratory biases that are independent of image content and task goals; and (3) in which reference frame these effects occur. Tactile stimulation to uncrossed and crossed hands during the viewing of static images resulted in long-lasting modulation of visual orienting responses. Subjects showed a well-known leftward bias during the early exploration of images, and this bias was modulated by tactile stimulation presented at image onset. Tactile stimulation, both at image onset and later during the trials, biased visual orienting toward the space ipsilateral to the stimulated hand, both in uncrossed and crossed hand postures. The long-lasting temporal and global spatial profile of the modulation of free viewing exploration by touch indicates that cross-modal cues produce orienting responses, which are coded exclusively in an external reference frame.

The selection of where we direct our gaze by moving our eyes and head depends on the interaction of multiple factors that can be divided into three major types: bottom-up or stimulus-driven, top-down or goal-driven, and spatial biases^1^. The decision process of how these aspects determine visual selection has been suggested to occur in a topographic ‘priority’ map into which these different factors are integrated^2^,^3^,^4^. Whereas bottom-up and top-down processes affect visual selection depending on the stimulus and task, spatial viewing biases, by definition, direct exploration independently of image content and task goals. For instance, humans tend to explore more of the center of the visual display while scanning complex scenes^5^,^6^,^7^ and are biased to start the exploration of a new stimulus predominantly on its left side^8^,^9^,^10^.

Most of the research on contingent factors of visual exploration has focused on the visual characteristics of the viewed scene. However, it is often necessary to direct gaze based on information from other senses like audition and touch. Both auditory and tactile cues can direct spatial attention^11^,^12^,^13^ and eye movements to precise locations^14^. These sensory modalities can also provide information about spatial locations at the far periphery and outside of the visual field and may thus extend spatial priority maps beyond what is accessible from only the visual domain^15^,^16^,^17^,^18^. Multimodal signals not only provide localized cues for covert attention and saccades, but they may, in addition, generate global orienting responses. Picture yourself, for example, on a hike through a forest. Songs of various birds may incite you to search the trees. The feeling of softness and obstacles under your feet, in contrast, may guide you to direct your gaze just ahead on your path to avoid stumbling. In these cases, sensory information does not direct gaze to its source. Instead, viewing behavior is biased more generally toward some region of the world, reminiscent of the above-mentioned leftward bias during free viewing. Such general orienting effects have been observed in several cross-modal tactile-visual covert and overt attention tasks. For example, tactile cues that are spatially uninformative result in faster responses to visual targets presented on the same side as the tactile stimulation, compared to targets presented in the opposite hemifield^19^. Similarly, tactile signals can effectively orient responses in real-life scenarios, for instance, as warning signals to avoid collisions while driving^20^. In free viewing, auditory information, too, can modulate exploration. When participants heard lateralized auditory information (e.g., bird-singing), their exploration of natural scene images was globally biased toward the side of the sound^21^,^22^. This effect of sound was additive to the effect of low-level visual saliency, consistent with the idea that auditory and visual information were integrated in a supramodal priority map.

In the present work, we focused on how tactile cues are integrated for the guidance of free-viewing behavior. The integration of spatial cues from touch can lead to conflict depending on the reference frame used to encode the tactile stimuli, a problem that does not apply to auditory stimuli. Touch is initially coded relative to the skin, for example, in the primary somatosensory cortex’s homunculus. However, because our body is flexible, the location of a touch in space crucially depends on the body’s posture at the time of touch. Therefore, the external touch location must be derived by integration of skin location and posture^23^. For experimental investigation, hand crossing allows dissociating the two reference frames. For instance, when the right hand (skin-based reference frame) is then located in left space (external reference frame). When a saccade is made toward a tactile stimulus on crossed hands, some saccades are initially directed toward the wrong hand but are corrected in flight^24^; for example, when the crossed right hand has been stimulated, the saccade starts toward the left hand that lies in right space, but ultimately lands at the correct (right) hand in left space. Similarly, a tactile cue can facilitate a visual decision when tactile and visual stimuli spatially coincide^25^. When the time interval between tactile cue and visual stimulus was short, facilitation occurred on the side to which the stimulated hand belonged, independent of hand crossing. When the time interval was long, however, facilitation occurred on the side of the space in which the stimulated hand was located^25^. Thus, facilitation appears to be guided initially by a skin-based reference frame, but later by an external one.

Yet, other findings suggest that the brain does not simply switch from using a skin-based to an external code, but that it retains the original, skin-based location and derives a location estimate by integrating both reference frames^26^,^27^,^28^,^29^. With crossed hands, tactile decisions can deteriorate, and such findings have been suggested to indicate a conflict in the integration of mismatching information stemming from different reference frames^23^,^30^,^31^. Accordingly, if touch can be integrated into a putative priority map in a similar way as audition, then the effect of touch may be mediated in a skin-based or in an external reference frame, or in both.

Here, we addressed three questions about the effect of tactile cues on visual selection. First, we evaluated whether tactile cues modulate eye-movement behavior in a general, modulatory way as shown before for auditory cues^21^,^22^. To this end, we provided random, task-irrelevant tactile stimulation to subjects’ hands (Fig. 1a,c) while they freely viewed complex scenes (Fig. 1b). Second, we addressed whether such a potential effect of touch would be restricted to viewing behavior that is contingent on stimulus content and task, or whether it could also modulate the leftward viewing bias that is present during early exploration of complex scenes^10^. Because it is known that visual exploration is spatially biased for some time after appearance of an image, we evaluated the effect of touch in two different time intervals. First, tactile stimulation was delivered *early*, at exactly 150 ms after image appearance. The effect of this *early* stimulation could be compared with the effect of touch *later* during image viewing (occurring randomly between 500 and 6000 ms after image appearance), when the exploration bias has subsided. Finally, by presenting tactile stimulation in two different hand postures, uncrossed and crossed (Fig. 1c), we evaluate the question of which spatial reference frame would underlie these effects of touch on visual selection.

## Results

**Figure 1:**
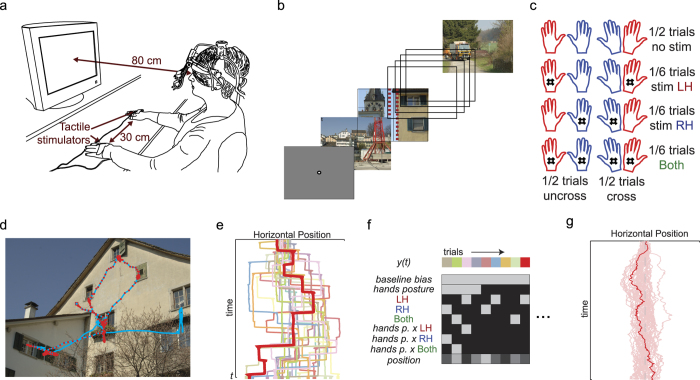
Setup and methods. (**a**) Illustration of the experimental setup in uncrossed hand posture. (**b**) Per block, 24 images were presented in succession. The first image appeared after a center drift correction. Afterward, image changes occurred after a minimum delay of 6 s and contingent upon a fixation in the horizontal center of the screen (red dashed lines, not shown during the actual experiment). (**c**) Schematics of hand crossing and early and late stimulation conditions. Stimulation at image appearance was presented in 192 of 384 trials (32 left, right, and bilateral for uncrossed and crossed postures). Late stimulation, between 0.5 to 6 s, was controlled by a random process such that the expected number of stimuli for each condition was the same as for early stimulation. (**d**) Example of one trial. The gaze scan path is shown in light blue for the complete period of 6 s; red dots are the actual gaze data for the first 3 s, sampled at 500 Hz, as used in the analysis. (**e**) Gaze horizontal position of the trial that is shown in red in (**d**); other traces are examples taken from other trials of the same subject. (**f**) Example of the design matrices used for the linear model. The dependent variable is gaze position for a given time point, and the independent variables are coded using a dummy scheme in which uncrossed hand posture and no stimulation are the reference conditions. The linear model is separately fitted for every time sample. (**g**) Example of the resulting baseline bias term at all time points for the subject (dark red) used as an example in previous panels and for the other 47 subjects that participated in the experiment (light red). Drawings in (**a**) and (**c**) by J. Ossandón and photographs in (**b**) and (**d**) by and with permission of S. Onat.

Forty-seven right-handed subjects participated in a free viewing task while their eye movements were recorded, and tactile stimulation was provided at different moments of visual exploration (Fig. 1a–c). Subjects were instructed to “study the images carefully” and told that the tactile stimulation was irrelevant to the task. The effect of tactile stimulation on subjects’ viewing behavior was evaluated at each point in time by fitting a general linear model of gaze position. The dependent variable was gaze position, and the independent variables were *baseline bias* (i.e., the model’s constant term), the presence of *tactile stimulation* (none, left hand, right hand, or bilateral), and *hand posture* (uncrossed or crossed). In the model, we also evaluated the interaction between *tactile stimulation* and *hand posture*, in order to disentangle skin-based from external coding, and a *position covariate* that took into account gaze position before the moment of stimulation (see an example of gaze data in Fig. 1d,e and a scheme of the linear model analysis in Fig. 1f,g). This analysis procedure resulted in time courses of the estimated model parameters over the duration of the image viewing trial. Four different sets of models were evaluated corresponding to biases in the horizontal and vertical dimensions during early and late stimulation events.

### Early exploration and the modulatory effect of touch

**Figure 2:**
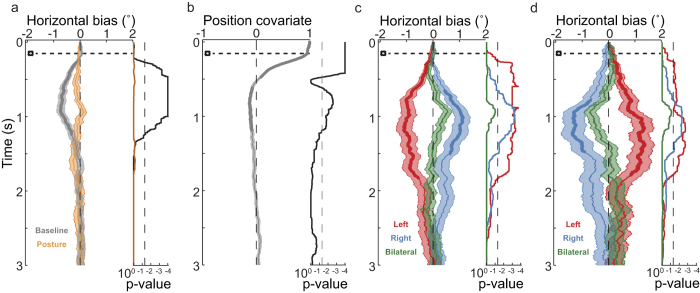
Results of the linear model for gaze horizontal position after image change (“early tactile stimulation”). Graphs show the progression of the different factors, and the light-color filled areas represent one standard error of the mean in each direction. Associated TFCE-corrected p-values are displayed on the right of each panel on a logarithmic scale. Significant periods are shown by thicker lines that indicate p-values below 0.05. After image start (time zero), the early tactile stimulation always occurred at 150 ms (horizontal dotted line). (**a**) Baseline bias and hand posture factors. (**b**) Covariate of gaze position before image change. (**c**) Tactile stimulation factor. (**d**) Interaction between tactile stimulation and hand posture.

We first evaluated whether subjects presented an early horizontal viewing bias^8^,^9^,^10^ in the absence of tactile stimulation. As expected for a right-handed sample, subjects exhibited a *baseline bias* to initiate exploration of new images on the left side. This bias is evident in Fig. 2a (gray line), which shows the progression in time of the constant *baseline* term of the linear model used to fit horizontal gaze position, corresponding to the condition of no tactile stimulation with the hands uncrossed. The constant term deviated significantly from the image midline in the interval between 270 and 1240 ms after image change. The effect of *hand posture* was not significant at any time point after image appearance. Therefore, hand crossing did not, by itself and in the absence of tactile stimulation, result at any time in a significant change in gaze position (Fig. 2a, orange line). Figure 2b shows the effect of the *covariate*, which corresponds to the gaze position prior to image change (not shown in the figure), and therefore starts with a value of 1 at the exact time of image change. Thus, the leftward *baseline* bias described above was independent of, and not explained by, the eye-position at the time of image change. The influence of the covariate was significant up to 474 ms (Fig. 2b), then changed sign and petered out in a small but significant effect in the opposite direction (616-1190 ms after image change). Thus, subjects showed a horizontal leftward bias during early exploration and tended to explore the contralateral side more than the ipsilateral region relative to their initial gaze position.

**Figure 3:**
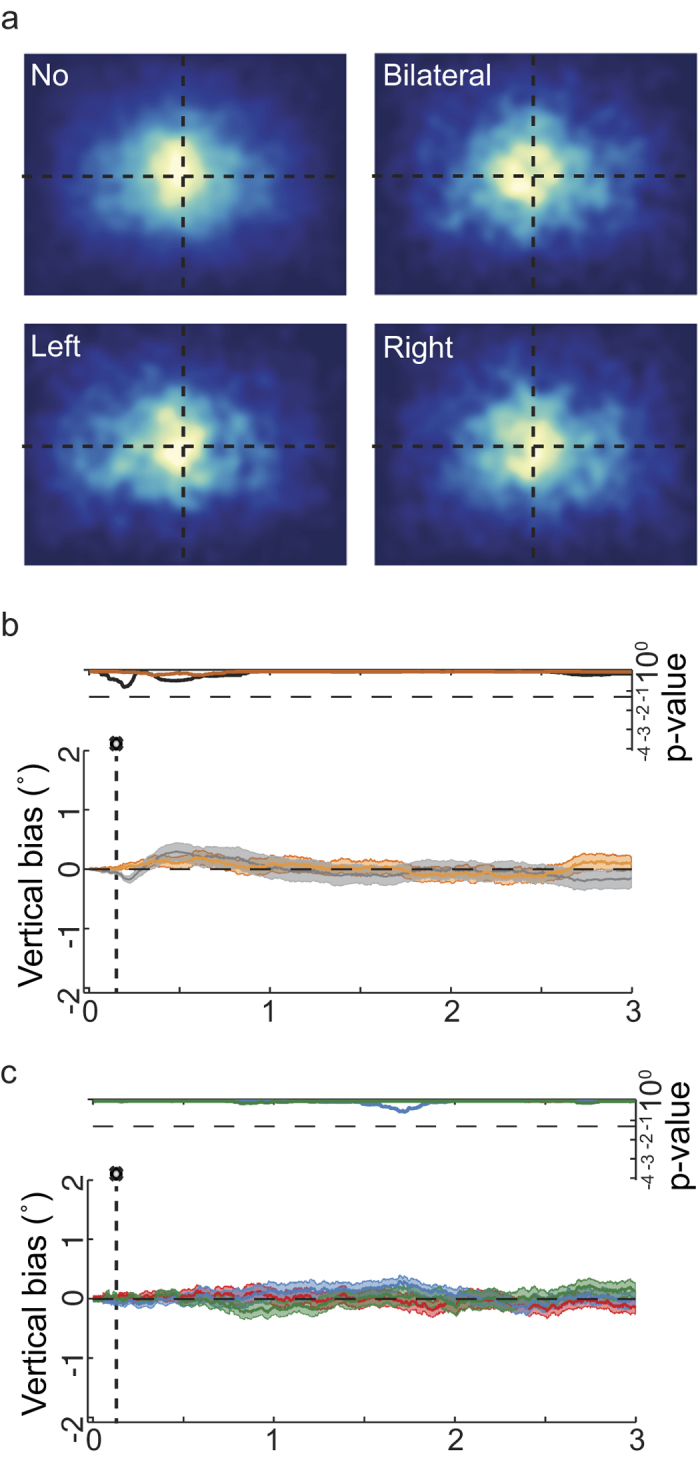
Effects of stimulation on the gaze vertical position (“early tactile stimulation”). (**a**) Fixation density map for fixation locations between 150 and 2000 ms after image change (stimulation occurs at 150 ms). (**b**) Baseline bias and hand posture factors for deviations of the gaze in the vertical dimension. (**c**) Tactile stimulation factor.

Next, we evaluated the effect of *tactile stimulation* to test whether touch modulated early visual exploration and the observed leftward bias. As the linear model included the interaction between factors *tactile stimulation* and *posture*, a significant effect of *tactile stimulation* indicates an additional bias directed to the stimulated hand in the uncrossed condition. Please note that, in this condition, anatomical and external coordinates are congruent, and we cannot differentiate between left-hand side and left side of space. The effect of tactile stimulation (indicated with a dotted line in Figs. 2 and 3), delivered always at 150 ms after image change (indicated as time zero in Figs. 2 and 3), resulted in an additional bias in the horizontal dimension that was directed to the space ipsilateral to the stimulated hand (Fig. 2c, *tactile stimulation*). The effect size was comparable for left and right tactile stimulation, although the effects on viewing behavior were more variable for right than for left stimulation across subjects. As a consequence, the touch-induced bias was significant from 108 to 1680 ms after left tactile stimulation, whereas it was significant from 656 to 1502 ms after right tactile stimulation. In contrast, bilateral tactile stimulation did not induce a significant bias. However, in Fig. 2c a tendency to the right after bilateral stimulation is apparent (lowest corrected *p-value* = 0.094). In summary, tactile stimulation produced a viewing bias that modulated the previously known, early leftward baseline bias by an additive component of comparable magnitude.

As a next step, we controlled for potential eye movements directly targeting the tactile stimulus. First, visual examination of the 2-D density of fixation probability over the images in the different stimulation conditions (Fig. 3a), evaluated in the interval between 150 and 2000 ms after image change, did not reveal a pattern of saccades directed downward or in the approximate direction from the midline to where the stimulated hand was positioned. Second, a linear model analysis of gaze position with vertical eye position as dependent variable, and with the same factors as in the analysis of horizontal image exploration, did not reveal any significant effects (see Fig. 3b,c). In summary, subjects did not perform targeted eye movements toward the region of the tactile stimuli on their hands. Consequently, the horizontal viewing bias induced by tactile stimulation was not a reaction towards tactile stimuli, but a true modulatory effect on image exploration.

The horizontal bias following tactile stimulation may be related to two different spatial reference frames. First, saccades may be biased toward the visual hemifield of the body side to which the tactilely stimulated hand belongs. Thus, right hand stimulation would lead to a right visual exploration because the right body side has received tactile stimulation (anatomical reference frame). Alternatively, saccades may be biased toward the hemifield in which the tactilely stimulated hand is currently located. Thus, right hand stimulation would lead to visual exploration of the right side of the image, because the right hand was located in the right side of space (external reference frame). Presenting tactile stimuli to crossed hands can disentangle these two accounts. If the effect of tactile stimulation were mediated in an anatomical reference frame, then the model’s interaction term of *tactile stimulation* with *hand posture* should not be significant and, accordingly, not modulate the viewing bias, given that we coded the side of tactile stimulation in anatomical space. In contrast, if the effect of tactile stimulation were mediated in an external reference frame, then the model’s interaction term should be significant and reverse the viewing bias expressed in the main effect of *tactile stimulation*. Indeed, the interaction between *hand posture* and *tactile stimulation* was significant (Fig. 2d) and resulted in a viewing bias pattern that was opposite to that of the factor *tactile stimulation* on its own (Fig. 2c): for left hand tactile stimulation, gaze tended to deviate toward the right in the crossed hand condition (that is, to the external location at which the hand was currently located), and vice versa for the right hand. Bilateral stimulation did not result in a significant bias, but again a trend is apparent in Fig. 2d (lowest corrected *p-value* = 0.08), this time directed to the left side (right-hand). This trend suggests that an orienting response directed to the external position of the dominant hand (here, the right) might exist when both hands are stimulated. However, as the model terms for bilateral tactile stimulation did not reach significance, and we did not test a group of left-handers, this interpretation is currently speculative. In summary, we found that early tactile stimulation biased free viewing behavior exclusively in an external reference frame.

### Effects of late tactile stimulation

**Figure 4:**
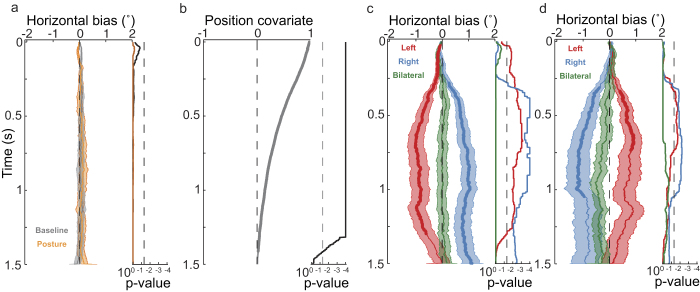
Linear modeling results for gaze horizontal position after stimulation during image viewing (“late tactile stimulation”). Graphs show the progression of the different factors, and the light-color filled areas represent one standard error of the mean in each direction. Associated p-values are displayed on the right of each panel in logarithmic scale. Significant periods are shown by thicker lines that indicate p-values below 0.05. Here time zero corresponds to the moment of stimulation, or in baseline no-stimulation hand-uncrossed trials, to the same times in which tactile stimulation occurred in stimulation trials. (**a**) Baseline bias and hand posture. (**b**) Covariate of gaze position before tactile stimulation (from −100 ms to the moment of stimulation). (**c**) Tactile stimulation factor. (**d**) Interaction between tactile stimulation and hand posture.

Examining the effect of *tactile stimulation* later during image exploration (occurring at random times from 500 to 6000 ms after image appearance) allowed us to evaluate cross-modal effects independent of viewing biases and other effects of a sudden image change event. Late stimulation resulted in a viewing bias pattern similar to that of early stimulation. However, by design, gaze location at the time of stimulation was different for early and late stimulation. Early stimulation was yoked to fixation around the image center (because presentation of a new image depended on this criterion). In contrast, because late stimulation occurred at random times, gaze could be at any position on the screen at the time of stimulation. Consistent with the fact that late stimulation did not coincide with an image change, the *baseline* term of the linear model (no stimulation and uncrossed hands) did not show any initial bias (see Fig. 4a, gray line). Similarly to the early stimulation results, the *hand position* factor did not reveal a bias (Fig. 4a, orange line). For almost the complete evaluated period of 1.5 s following tactile stimulation, the covariate of pre-stimulation gaze position resulted in a strong centering effect (Fig. 4b) that simply indicates that the further current gaze position was from the image’s center, the more likely the gaze was directed toward the image center. All other factors of the linear model showed a similar pattern of results as those for early tactile stimulation. *Tactile stimulation (time zero in* Fig. 4) biased exploration in the direction of the stimulated hand in the uncrossed condition, from 31 to 1300 ms for left stimulation, and to the right from 277 ms to the end of the evaluated period (1500 ms) for right stimulation (see Fig. 4c). Bilateral stimulation did not result in a significant bias. The interaction between hand posture and stimulated hand reversed the pattern for the viewing bias observed for *tactile stimulation* (Fig. 4d) in the crossed posture condition, indicating that touch affected free viewing in an external reference frame. Stimulation of the crossed left hand resulted in a gaze bias to the right from 337 to 733 ms, and stimulation of the right hand resulted in a gaze bias to the left from 311 to 1047 ms. As for early stimulation, we did not observe any significant effects of tactile stimulation on vertical saccade direction (data not shown), indicating that for late stimulation, too, biases were not due to reflective orienting towards the stimulated hand. In summary, just as early tactile stimulation did, late tactile stimulation of the hands biased visual exploration, indicating that the induced viewing bias did not depend on gaze being centered on the screen.

### Effects of stimulation and image change in saccade programming

Tactile stimulation could affect saccade programming at different stages. Reorientation of the direction of a saccade could occur when it is already on course^24^, by changing the spatial target of a saccade that is already being programmed, or by entirely resetting any potentially ongoing saccadic programming. The current experimental design prevented analysis of in-flight saccade redirection because explicit saccade start and end points would have to be known in order to assess deviations from a default trajectory, but these are not available in a free-viewing task. We were able, however, to evaluate the resetting option by evaluating saccade latencies after tactile stimulation. Because image change has an effect on saccade re-programming^10^, any re-programming effect of tactile stimulation cannot easily be disentangled from the effect of image change at the beginning of a viewing trial. Instead, we analyzed latencies after late stimulation during image viewing.

**Figure 5:**
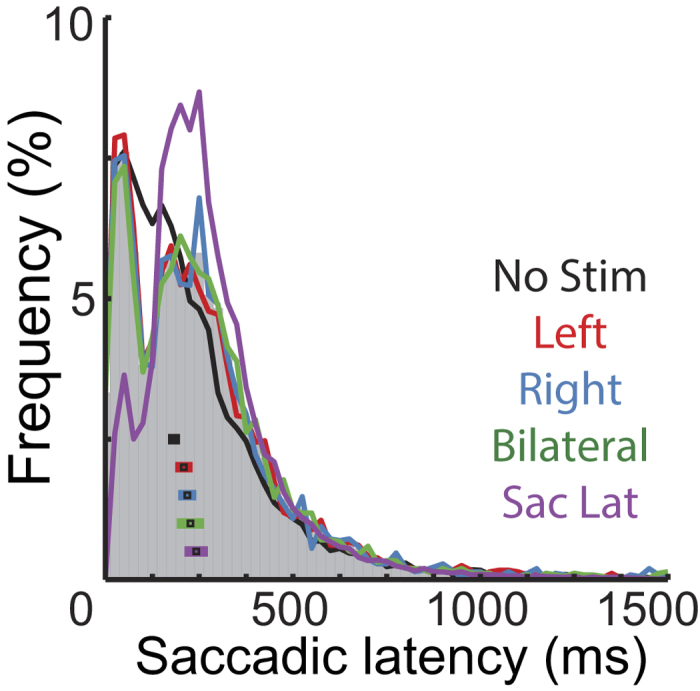
Distributions of saccade latencies after tactile stimulation. The bar histogram displays the grand total distribution of saccadic latencies of all stimulation conditions. The curves display data of different conditions; black squares within the histogram correspond to conditions’ median values plus/minus the absolute median deviation. Two control distributions were used to evaluate saccadic resetting: the no stimulation control (black, No Stim) corresponds to latencies of saccade onsets in trials without stimulation sampled at the same times as stimulation occurred in stimulation trials. Saccadic latency control (purple, Sac Lat) corresponds to the normal latency of saccades (i.e. fixation durations).

The distribution of saccade latencies after tactile stimulation (i.e. the time from stimulation to movement) was bimodal with an early peak at 45 ms, a second peak at 165 ms (Fig. 5), and the antimode at 105 ms. This distribution strongly differed in pattern from a control distribution constructed with saccade latencies in no-stimulation trials, sampled at the same times stimulation occurred in stimulation trials (see Fig. 5, black trace; χ^2^ goodness of fit test, comparison between no-stimulation control and pooled stimulation conditions: χ^2^_(61)_ = 304.5, *p* < 0.0001). Distributions median values were also significantly different (No/left, *z* = −4.01, *p* < 0.0001; No/Right, *z* = −4.63, *p* < 0.0001; No/Bilateral, *z* = −4.44, *p* < 0.0001). Stimulation conditions did not differ from each other (all *p* > 0.05). This result suggests that the programming of saccades was affected by the occurrence of a tactile stimulus. Yet, if tactile stimulation always resulted in a resetting of saccade programming, the distribution of latencies should resemble a shifted normal saccadic latency (i.e. fixation durations) compared to when no stimulation occurred (Fig. 5, purple line). However, this distribution also had a different shape from the stimulation conditions (normal saccadic latency against pooled stimulation conditions: χ^2^_(61)_ = 1308.6, *p* < 0.0001) as well as a higher median value (No/left, *z* = −3.74, *p* = 0.0002; No/Right, *z* = −3.13, *p* = 0.0009; No/Bilateral, *z* = −2.38, *p* < 0.017), suggesting that, although touch affected saccade programming, it did not always induce reprogramming. This result is in line with the finding that a programmed saccadic movement cannot be stopped later than 124 to 145 ms before it starts^32^. Therefore, tactile stimulation occurring in this final interval of the programming of a saccade presumably could not stop an already triggered movement. The most plausible explanation for the bimodal shape seen here is, therefore, that tactile stimulation resulted in a resetting of saccadic programming, but left unaffected a population of saccades that were already beyond their stopping point and that are represented by the first mode of the distribution.

## Discussion

We showed that task-irrelevant tactile stimulation resulted in a long-lasting free viewing bias. This bias was directed globally to the images’ side ipsilateral to the location of stimulation in external coordinates. It was present both during the initial period of image viewing in which there was a strong horizontal spatial bias, as well as during later viewing which was dominated by contingent, content-based, exploration of the images.

Tactile stimulation has previously been shown to affect the orienting of spatial attention in covert and overt attention tasks. In contrast to previous research, we presented a description of the effect of touch during free viewing behavior, which is closer to how we explore the visual world outside of experimental settings. The fine-grained spatial and temporal analysis used here allowed us to investigate in which reference frame touch affects free viewing. Tactile location has been demonstrated to be represented both in skin-based anatomical and external reference frames in a wide variety of tasks^23^,^25^,^33^,^34^,^35^, including when the touch is the target of an eye movement^24^,^36^,^37^. In contrast, the biasing effect of tactile stimulation on visual exploration in the present study was exclusively coded in an external reference frame.

Although many studies have reported effects of both anatomical and external reference frames, several studies suggest that, in some tactile tasks, behavior is predominately modulated by an external reference frame^13^,^38^,^39^. Such variability in the use of different reference frames is consistent with the suggestion that spatial location in touch is derived by weighted integration of information from different reference frames and may be highly task-dependent^26^,^28^,^29^. In fact, whereas studies in which subjects were required to make saccades to tactile targets have reported the use of both anatomical and external reference frames^24^,^36^,^37^, a study in which touch co-occurred with saccades but was not the saccade target reported that the enhancement of touch by saccade planning was mediated in an external reference frame^40^. Here, we investigated the reverse link of an influence of task-irrelevant touch on saccade planning and again observed the use of only an external reference frame. Yet, the idea that only the external reference frame is relevant whenever touch is not task relevant cannot be generalized, as other paradigms that did not involve saccades have reported mixed reference frame use for task-irrelevant touch^41^.

The fact that anatomical guidance occurred only for short-latency saccades in a previous study^24^ led us to expect that we should find an anatomical bias for saccades occurring early after tactile stimulation and an external effect for saccades occurring later. Analysis of late saccades in the present study suggested that saccade planning was reset by tactile stimulation, suggesting that saccades directed to tactile stimuli^24^ and saccades merely evoked by tactile stimulation (present study) should be readily comparable. However, we did not observe a change of reference frames in the time course of the viewing bias with respect to hand posture, suggesting that, in fact, the reference frame used to guide the viewing bias in natural scenes was selectively external. Yet, incorrectly directed saccades occurred only infrequently in Overvliet and colleagues’ study even for early saccades. Therefore, it appears possible that some saccades in the present study, too, were modulated in an anatomical reference frame, but that such an influence may not have been observable in our bias measure that aggregated over a large number of saccades.

The external spatial effect of touch on visual selection lasted for several seconds, that is, it affected several saccades that followed tactile stimulation. This temporal profile is reminiscent of endogenous attentional orienting, which is characterized by late latency, long-lasting orienting responses to non-local symbolic cues^42^,^43^,^44^,^45^. In comparison, exogenous cueing is said to be characterized by short-latency, short-lived responses to peripheral local cues^42^,^43^,^44^,^45^,^46^. In the present study, the tactile stimulus was spatially localized, and it was non-symbolic. However, its effects were spatially generalized, directed toward one side of space, and not restricted to the location of the touch. This generalized spatial orienting effect is also characteristic of endogenous responses and similar to the effect of auditory cues during free viewing^21^,^22^. Together with the temporal profile of the tactilely induced bias, the current results therefore suggest that touch was integrated like an endogenous attentional cue, rather than as an exogenous one.

In our experiment, tactile stimulation was unrelated to the task and occurred outside the defined spatial area for exploration, with the hands placed underneath the monitor. This setting probably resulted in an internal framing of spatial attention, which is a mechanism suggested for the absence of capture by salient external events when they are localized elsewhere^47^,^48^. It is therefore possible that we did not observe saccades to the location of tactile stimulation because the hands were outside subjects’ window of attention. This may also be a relevant factor for the occurrence of tactile orienting in a purely external reference frame in the present study.

To conclude, our results demonstrate that exploratory viewing behavior can be spatially biased for extended periods of time by short, irrelevant tactile stimuli. As sound can also bias free viewing in a similar, spatially generalized manner, our results suggest that non-visual stimuli can exert surprisingly extensive biases on spatial orienting behavior when the perceived stimulus is not selected as a direct target for visual fixation. Together with previous research, our results support the idea that visual selection is based on the integration of multisensory local cues, global orienting signals, and of non-sensory bias signals, possibly in some type of unique priority map^2^,^4^,^37^,^49^.

## Methods

### Participants

Forty-seven right-handed subjects (29 females; mean age: 21.8 years; range: 18-29; SD: 2.3) with normal or corrected-to-normal vision participated in the study. Handedness was evaluated with the Edinburgh handedness inventory^50^. In all experiments presented in this article, written consent was obtained from each participant, and the experimental procedure were carried out in accordance with the Declaration of Helsinki and approved by the ethics committee of the University Osnabrück.

### Stimuli

Following previous work^10^,^51^, four different kinds of visual stimuli were used: The first category (naturals) included 64 scenes taken from the Calibrated Colour Image Database^52^ depicting outdoor scenes without man-made objects or buildings. The second category (man-made) included 192 urban scenes, 64 of them from public spaces around Zürich taken with a high resolution camera (Nikon D2X) by one of our co-workers (S. Onat), and 128 taken from the LabelMe database^53^. Urban scenes did not include text. The third category included 64 fractal images. The fourth category included 63 1/f noise color images that were generated from the images of the three other categories: Images belonging to each category were transformed to the Fourier space, and their average power spectra were combined with random phases taken from a uniform distribution. The complete image set was duplicated by mirror reversing the images. Each subject was presented with only one version of each image. Image category as a factor did not result in significant effects in the linear model explained below and was therefore excluded from the models we report here.

Images were presented at a distance of 80 cm on a 21” CRT monitor (Samsung SyncMaster 1100 DF, Samsung Electronics, Suwon, South Korea) at a refresh rate of 85 Hz and a resolution of 1280 × 960. The active part of the screen subtended a visual field of 28° horizontally and 21° vertically. One visual degree was equivalent to 45.6 pixels.

### Eye-tracking

Eye movements were recorded with a head-mounted video oculographic eye-tracking system using binocular pupil tracking at 500 Hz (Eyelink II, SR Research Ltd., Mississauga, Canada). Eye position was calibrated with a 3 × 3 grid until the average measurement error was below 0.5°. Fixation locations were calculated by the eye-tracker using the system default parameters, defining fixation periods as the complement of blink and saccade events. Saccades were defined from a minimum deflection threshold of 0.1°, a velocity threshold of 30 °/s, and an acceleration threshold of 8000 °/s^2^.

### Tactile stimulation

Tactile stimulation consisted of vibratory stimulation at 200 Hz for a duration of 50 ms to the back of subjects’ hands. It was produced by Oticon bone conductors (Oticon Ltd, Milton Keynes, UK, part number 461-012, size about 1.6 × 1.0 × 0.8 cm). Stimulators were controlled by custom-built hardware triggered through the parallel port for millisecond-precision timing. Subjects wore earplugs to block the noise of the tactile stimulators.

### Procedure

Experiments comprised 394 images and were completed in single sessions of approximately one-hour duration. Figure 1a depicts the setup. Subjects sat in front of the monitor in a darkened room. Their hands were placed comfortably on a table in front of them in either an uncrossed or a crossed (left over right) posture. The distance between the two hands was 30 cm in all conditions. Images were presented in 16 blocks of 24 images each. Eye tracker drift correction was performed before the first image of a block, and the first image was never analyzed (see Fig. 1b for a description of one block). Eye-tracker calibration was renewed every other block. Hand posture was altered every 4 blocks, with the starting posture balanced across subjects. Before the experiment, a training block of 10 trials was run, but not analyzed.

Each image was presented for at least 6 s. After this period, the appearance of the next image was contingent upon subjects’ gaze position: the next image was presented after a fixation had begun in an area inside 6° around the images’ vertical meridian. Concurrent with the free viewing task, tactile stimulation was delivered at different moments. When *early* stimulation occurred, it was always delivered at 150 ms after an image change. *Late* stimulation could occur randomly at any moment between 500 ms after an image change and the end of the trial (6 s).

Early tactile stimulation was presented randomly in only half of all trials, either on the left, on the right, or bilaterally (Fig. 1c). To ensure that *late* stimulation was also unpredictable, ISI were sampled from an exponential distribution with a minimum value of 500 ms and a median equal to the trial duration of 6 s, resulting in a constant hazard function. Accordingly, a tactile stimulus occurred in only half of the trials on average and with the same probability of being presented either to the right, the left, or both hands. Thus, for both early and late stimulation, there were 32 stimuli for each combination of stimulus location (left, right, bilateral) and hand posture (uncrossed, crossed).

### Data analysis

The effects of stimulation were evaluated by applying a linear model to the dependent variable gaze position for every gaze sampling point. Two mass-univariate models were calculated for each subject at every gaze sample, one to evaluate effects occurring after image change and *early* stimulation (from 0 to 3 s after image change and stimulation, 1500 samples) and the other to evaluate effects of *late* stimulation that occurred later during image exploration (from the moment of stimulation to 1.5 s after stimulation occurred, 750 samples). The temporal extent of the analysis window for late stimulation was reduced to 1.5 s as a compromise between having a period of analysis long enough to include several eye-movements after stimulation, and thus allowing to detect long-term biases, with also being able to include as many possible occurrences of late stimulation, considering that stimulation occurred at random times during the interval between 0.5 and 6 seconds and that only complete periods of time after stimulation were to be included in the analysis. Thus, we effectively analyzed viewing biases for tactile stimuli occurring between 0.5 and about 4.5 s after image change. Dummy coding was used to evaluate the influence of main factors *tactile stimulation* and *hand posture* against the reference group (constant term) of no stimulation and hands uncrossed. The interaction of *tactile stimulation × hand posture* was included to address the possible effect of stimulation in different reference frames. Finally, we included the average gaze position before stimulation, relative to the midline, as a covariate. For early stimulation, we used the 100 ms before an image change, as this gaze position triggered the new image. For late stimulation, we used the 100 ms directly preceding stimulation. In the case of the *late* model, the reference group of values of no stimulation was taken randomly from trials without stimulation, at the same moments tactile stimulation occurred in the stimulation trials.

Second-level analyses were performed across subjects by testing the regression coefficients associated with each factor and time sample with a one-sample t-test against the null hypothesis of no effect. This results in 1500 (early stimulation) and 750 (late stimulation) t-tests for each factor. To control for the possible elevated familywise error rate (FWER), we calculated threshold-free cluster enhancement values (TFCE)^54^ for each sample and factor and compared them to an appropriate null distribution of control values. TFCE calculates the cluster-like local temporal support of every t-value and is given by the sum of all sections in time that are underneath it. This local temporal support is the sum of all samples that are not beyond and higher than any local minima that is between them and the sample under calculation. For the gaze position data, this is calculated as follows:
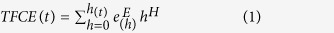


For every gaze sample (t), the TFCE value equals the sum of the score of all sections underneath it, which is in turn defined by its height (*h* in t-statistic units) multiplied by its extension (*e* in sample units). Extension and height are raised to the power of parameters E = 0.5 and H = 2 respectively^54^. TFCE values were calculated separately for positive and for negative *t*-values and grouped together afterwards as only positive values. TFCE values for each factor and sample were compared against a null distribution obtained by taking the maximum TFCE values of 1,000 permutations of coefficients sign across subjects.

## Additional Information

**How to cite this article**: Ossandón, J. P. *et al*. Irrelevant tactile stimulation biases visual exploration in external coordinates. *Sci. Rep*. **5**, 10664; doi: 10.1038/srep10664 (2015).
